# The Cytoskeleton and Its Binding Proteins as Mechanosensors, Transducers, and Functional Regulators of Cells

**DOI:** 10.3390/ijms25010172

**Published:** 2023-12-22

**Authors:** Wan Lee

**Affiliations:** 1 Department of Biochemistry, Dongguk University College of Medicine, 123 Dongdae-ro, Gyeongju 38066, Republic of Korea; wanlee@dongguk.ac.kr; Tel.: +82-54-770-2409; Fax: +82-54-770-2447; 2 Channelopathy Research Center (CRC), Dongguk University College of Medicine, 32 Dongguk-ro, Ilsan Dong-gu, Goyang 10326, Republic of Korea

## 1. Introduction

Due to its complement of diverse proteins, such as actin filaments, intermediate filaments, and microtubules, the cytoskeleton is essential not only for structural stability but also for regulating cellular signaling, intracellular transportation, and cell division [1,2,3]. Moreover, cytoskeleton-binding proteins (CBPs) orchestrate these functions by interacting with cytoskeletal components to form a complex system key to generating cellular biochemical responses to physical and mechanical stimuli through mechanosensing and mechanotransduction [4,5]. Therefore, the dysregulation of these interactions can lead to numerous diseases, such as malignancies, degenerative disorders, and metabolic diseases [6,7,8].

During recent decades, researchers have endeavored to decode molecular interactions between the cytoskeleton and CBPs [2]. Diverse biochemical and biophysical analyses have been employed to investigate the mechanical properties of CBPs and their responses to intracellular stimuli [9,10]. Moreover, advanced imaging techniques, including super-resolution microscopy and live-cell imaging, have provided a greater understanding of the dynamics of these proteins [11,12]. Despite considerable advances, challenges persist regarding the comprehension of the intricate interactions and regulatory mechanisms of the cytoskeleton and CBPs. Unraveling the roles of cytoskeletal dynamics and the signaling pathways involved in mechanotransduction is crucial for developing innovative therapeutic approaches targeting these components in various health conditions and disease backgrounds. This Special Issue, entitled “Cytoskeleton and Its Binding Proteins as Mechanosensors, Transducers, and Functional Regulators of Cells” and published in the *International Journal of Molecular Sciences*, includes five significant articles (contributions 1–5) that advance our understanding of cytoskeletal biology and mechanotransduction and highlight potential therapeutic avenues.

## 2. Exploring This Special Issue

Accumulating evidence has highlighted the importance of actin cytoskeleton dynamics in skeletal myogenesis, as influenced by the interplay between CBPs and the Hippo signaling pathway. In the first article by Nguyen et al. (contribution 1), the authors reveal the critical function played by actin-binding protein FLII during myogenic progenitor cell differentiation. FLII levels are downregulated during the myogenesis of C2C12 myoblasts, and the authors found that FLII knockdown in myoblasts led to the upregulation of filamentous actin (F-actin), the nuclear translocation of YAP1, and the activation of genes crucial for cell proliferation. Subsequent experiments revealed that FLII knockdown inhibited the expressions of myogenic regulatory factors, hindering myoblast differentiation and myotube formation. The crucial role of FLII in the regulation of the F-actin/YAP1 axis during myogenic differentiation is highlighted in this study, which suggests that FLII could be a feasible therapeutic target for muscle wasting.

In the second article, Kazuo Katoh (contribution 2) discusses the effect of mechanical stress on endothelial cells, which are essentially required for vascular homeostasis. The review explains the basic principles of the cellular response to mechanical stresses and explores how these mechanical stresses affect endothelial cells in vitro and in situ. The article also introduces the signal transducers in the mechanotransduction mechanisms regulated by cytoskeletal components in the endothelial cells. Understanding the impact of mechanical stress on endothelial cells is crucial for comprehending complex interactions within the vascular system in health and disease. It will thus provide valuable insights into vascular physiology and pathology.

In the third article, Coscarella et al. (contribution 3) comprehensively review the role of mechanotransduction in cardiomyocytes and its implications in cardiomyopathies. The authors explore the crucial communication between the cytoskeleton and the nuclear envelope, which changes nuclear remodeling, gene expression, and its involvement during various pathogenic processes. The review also discusses molecular features in cardiomyocytes, and especially how signals from sarcomeric contractions are transmitted to different cellular parts and impact gene expression. Also, the authors connect dysfunctional sarcomeric roles and contractility in cardiomyocytes with inherited or acquired sarcomeric variants. The conclusions of this comprehensive review emphasize further research on mechanotransduction and nucleus enveloping dynamics in cardiomyocytes to understand the pathophysiology of cardiomyopathies.

In the fourth article, Sarantelli et al. (contribution 4) explore the significance of the role of Fascin-1 in the migration and invasion of cancer cells and offer pathways to novel anti-metastatic therapeutic targets. Fascin-1, an actin-bundling protein, plays a crucial role in cell migration, which is essential in physiological and pathological contexts, including cancer metastasis. Throughout the article, the authors analyze the latest research on Fascin-1, with a particular focus on its expression in various types of cancer, its role in altering the mechanical properties of cancer cells, and its role in promoting the migration, invasion, and metastasis of cancer cells. Furthermore, the review discusses the potential effects of inhibiting Fascin-1 in vitro and in vivo using a variety of pharmacological agents on metastasis and the fact that Fascin-1 is a crucial component of metastasis, highlighting its potential as an important anti-metastatic target that deserves further investigation. 

In the last article, Cornelison et al. (contribution 5) address the urgent need for effective treatments for this disease. Rhabdomyosarcoma is the most common pediatric soft-tissue malignancy, with a low survival rate for high-risk children. The authors place focus on the novel oncogenic actin-binding protein AVIL, which is commonly overexpressed in rhabdomyosarcoma. The review focuses on the significance of the actin-binding protein AVIL, as identified by studies on rhabdomyosarcoma cell lines, patient-derived xenograft models, and clinical samples of the alveolar and embryonal subtypes. The authors propose that AVIL be viewed as a therapeutic target and emphasize its potential ability to enhance the efficacy and specificity of cytotoxic agents. They suggest that this approach offers promise for the effective, safe treatment of this challenging pediatric cancer.
